# Residential greenspace and multiple chronic health conditions in China: a cross-sectional study

**DOI:** 10.7189/jogh.15.04218

**Published:** 2025-07-25

**Authors:** Siyuan Wang, Jingjie Sun, Zhiwei Xu, Gian Luca Di Tanna, Mingsheng Chen, Laura E Downey, Stephen Jan, Lei Si

**Affiliations:** 1 The George Institute for Global Health, University of New South Wales, Sydney, Australia; 2 Shandong Health Commission Medical Management Service Center, Jinan, China; 3 School of Medicine and Dentistry, Griffith University, Gold Coast, Australia; 4 Department of Business Economics, Health & Social Care, University of Applied Sciences and Arts of Southern Switzerland, Lugano, Switzerland; 5 School of Health Policy and Management, Nanjing Medical University, Nanjing, China; 6 Jiangsu Health Vocational College, Nanjing, China; 7 Imperial College London, Faculty of Medicine, London, UK; 8 School of Health Sciences, Western Sydney University, Sydney, Australia; 9 Translational Health Research Institute, Western Sydney University, Sydney, Australia

## Abstract

**Background:**

Multiple chronic conditions are imposing an increasing health and economic burden on the Chinese health system. While exposure to residential greenness has been shown to provide various health benefits, its relationship with multiple chronic conditions remains largely unexplored. This study aims to investigate this relationship using high-resolution satellite imagery and data from the 6th Health Services Survey (HSS) cohort in Shandong province.

**Methods:**

We linked health data from the HSS with 12-month average Normalised Difference Vegetation Index (NDVI) measurements based on reported residential geocodes. Multiple chronic condition status was defined as having two or more chronic conditions concurrently, according to the HSS’s predefined disease classification. Generalised mixed regression models were utilised to assess both the likelihood and count of multiple chronic conditions in relation to greenspace exposure. Additionally, using the pre-defined disease classes, we also explored how greenspace influences multiple chronic conditions across various physiological systems and disease categories

**Results:**

A total of 28 489 individuals were included in this cross-sectional analysis. After adjusting for potential confounding factors, we found that exposure to greenspace was significantly associated with a reduced prevalence and count of chronic conditions. Adjusted odds ratios (aORs) and 95% confidence intervals (CIs) for were: Q2 (aOR = 0.74; 95% CI = 0.62, 0.88), Q3 (aOR = 0.69; 95% CI = 0.55, 0.86), and Q4 (aOR = 0.70; 95% CI = 0.56, 0.88), respectively, compared against the baseline Q1 quartile. Subgroup analyses revealed that higher residential greenspace exposure reduced risks of blood, endocrine, nutritional and metabolic chronic diseases. No clear associations were found for other chronic disease classes. Additionally, consistent results were observed across spatial and temporal sensitivity analyses.

**Conclusions:**

Our findings underscore the potential beneficial effects of residential greenness on multiple chronic conditions, with implications for urban planning, environmental policy, and community development.

Multiple chronic conditions, or multimorbidity, refer to the coexistence of two or more chronic health conditions within an individual [1]. Individuals with multiple chronic conditions often report poorer health outcomes, including reduced quality of life, shorter life expectancy, and higher health care expenditures, compared to those managing a single chronic disease [2–4]. Multiple chronic conditions pose significant challenges to health care systems due to the complex and multifaceted care needs required for patients [5,6]. This is particularly evident in low- and middle-income (LMICs) settings, where health care systems have traditionally been vertical and single-disease focused [7,8]. In China, the Centre for Disease Control and Prevention reported that an estimated 46.5% of Chinese adults aged 18 years and over were living with more than one chronic health condition in 2018 [9]. Among individuals over 45, this figure rises to over 57% [10]. Additionally, researchers have predicted a substantial future increase in the prevalence of multiple chronic conditions in China, driven by an aging population and elevated levels of risk factors for non-communicable diseases (NCDs) [11,12].

Evidence has shown that exposure to residential greenspace, which refers to the surrounding vegetation in a neighbourhood, can improve mental health outcomes, enhance overall perceived health and reduce mortality risks associated with chronic NCDs such as cardiovascular diseases, chronic respiratory diseases and mental illnesses [13–17]. While the exact mechanisms underpinning this beneficial association is still largely unknown, several possible behavioural and ecological pathways have been identified. Exposure to residential greenness can reducing stress, promoting physical activity, improving air quality, and providing opportunities for social interactions, all of which can potentially contribute to this positive relationship [18,19]. Existing research has primarily focused on assessing the relationship between exposure to residential greenspace and individual chronic conditions, often overlooking that individuals with multiple chronic conditions frequently share common risk factors and underlying disease aetiologies. Further, the potential mechanisms through which greenspace benefits overall well-being involve multiple physiological systems and interrelated pathways. Thus, investigating the relationship between greenspace and multiple chronic conditions is warranted, given the potential cross-system, multi-organ benefits it exerts on human health.

We utilised high-resolution satellite data to investigate the relationship between residential greenspace and multiple chronic conditions. We hypothesised that greater exposure to greenspace would be associated with decreased odds of experiencing multiple chronic conditions.

## METHODS

### Study design and population

We conducted a cross-sectional analysis using data from the 6th Health Services Survey (HSS) from Shandong province. The HSS is a provincial-level health census survey that has been conducted in Shandong province every five years under the commission of the government. It contains individual-level data on socioeconomic status, health care utilisation, quality of life, and other self-reported health outcomes. Specifically, the 6th HSS employed a multi-stage cluster random sampling method. Twenty districts within Shandong province were selected, and within each district, two villages were sampled from each of the five townships, resulting in 10 villages per district. In each village, 60 households were selected, totalling approximately 12 000 households. Face-to-face interviews were conducted by trained professionals between September and October 2018 using a structured questionnaire. In total, 35 262 individuals from 12 938 households were interviewed across more than 150 counties or cities.

### Health outcome

Multimorbidity status was determined based on self-reported medical diagnosis where respondents were asked whether they had received a diagnosis from a medical professional or undergone treatment for chronic conditions within the past six months. Conditions were classified from the questionnaire’s prespecified list and include chronic conditions such as: diabetes, hypertension, cardio-cerebrovascular disease, cancer, mental and cognitive illnesses, chronic respiratory diseases, arthritis and other chronic conditions (Table S1 in **Online Supplementary Document**). Individuals were assessed as having multiple chronic conditions if there were two or more chronic conditions present. Additionally, we also quantified the number of chronic conditions for individuals as a count variable from 0 to 5.

### Environmental exposure

We utilised Normalized Difference Vegetation Index (NDVI) measured from the Terra Moderate Resolution Imaging Spectroradiometer Vegetation Indices (MOD13Q1), with a 250m*250m spatial resolution, measured every 16-day, as a representation for residential greenness. Normalized Difference Vegetation Index is a remote sensing measurement used to assess vegetation density, calculated by comparing the difference between near-infrared and red light reflected by the Earth's surface [20]. It is a commonly used numerical indicator that assesses vegetation density on a scale from 0 to 1, where values closer to 1 indicate a higher density of vegetation cover [20]. Vegetation density around each respondent’s reported geo-coded location was measured using annual NDVI values within a 1 km radius buffer (NDVI 1000 m), serving as a representation of long-term greenness exposure [20]. Furthermore, in order to conduct sensitivity analyses, we also computed NDVI values for 2 km, 3 km, 4 km, and 5 km radius buffers, along with 24-month and 36-month averages preceding October 2018.

### Covariates and mediators

We accounted for potential individual-level confounders by adjusting for several key variables including age (below 45, 45 to 65, and above 65), gender (male or female), education (<7 years of education, 7 to 9 years of education, and >9 years of education), region (urban or rural), marital status (never married or married and other), annual household income reported in Chinese Yuan (CNY) (≤40 000 CNY and >40 000 CNY, where 40 000 CNY was the reported median), and body mass index (BMI) (<18.5, 18.5, 24.9, and ≥25).

### Statistical analyses

We conducted a descriptive analysis of our sample population. The χ^2^ test was utilised to examine the disparities of respondents with and without multiple chronic conditions stratified by exposure to residential greenness (measured by NDVI) and individual-level covariates, including age, gender, marital status, region, education, income, and BMI. Normalized Difference Vegetation Index values were divided into quartiles to account for the nonlinear relationship between exposure to residential greenness and multimorbidity status. We utilised Generalized Linear Mixed Models (GLMM) with village-level random effects to assess dose-response relationships. GLMMs are commonly employed in similar cross-sectional settings to account for variability among different sampling clusters. Specifically, we adjusted for several covariates including age, gender, marital status, region, education, income, and BMI. Additionally, a Poisson regression with random effects was utilised to model the association between the count of chronic diseases and exposure to greenspace, considering for all covariates.

Sub-group analysis was conducted to investigate the association between exposure to residential greenspace and various chronic diseases clusters. Disease groups were chosen from the prespecified classes from the HSS (Table S1 in **Online Supplementary Document**) including metabolic diseases, mental and cognitive diseases, cardiovascular diseases (CVD), respiratory diseases, musculoskeletal diseases, digestive system diseases, and genitourinary system diseases. We adjusted for covariates including age, gender education, income, marital status, region, and BMI. Additionally, we conducted a series of sensitivity analyses to assess the reliability of our findings. In addition to the 12-month NDVI measurements, we incorporated average NDVI data from 2016 and 2017 to account for potential delayed effects of greenspace exposure on various chronic conditions. Second, we repeated our analysis utilising radius buffers of 2 km (NDVI_2000m_), 3 km (NDVI_3000m_), 4 km (NDVI_4000m_), and 5 km (NDVI_5000m_). Lastly, we stratified our sample cohort by gender, age, and region to assess the consistency of greenspace health effects across different demographic groups. We reported our findings in accordance with the STROBE guidelines for cross-sectional studies (Table S2 in **Online Supplementary Document****)**.

## RESULTS

### Descriptive analysis

A total of 28 488 respondents were included in this cross-sectional analysis (Figure S1 in **Online Supplementary Document****)**. **Table 1** reports the baseline characteristics of the sample cohort. The median age of the cohort was 50 years and interquartile range (IQR) of 26 years. Average annual household income median was 40 000 CNY, (IQR = 40 000 CNY), and median BMI was 23.7 kg/m^2^ (IQR = 4.9). Additionally, Quantile-quantile (QQ) plots and histograms for each variable were plotted, suggesting that the distributions of age, income, BMI, and NDVI were non-symmetrical (Figure S2–5 in **Online Supplementary Document**). Furthermore, 2308 respondents (6%) reported experiencing more than one chronic condition. Participants with multiple chronic conditions were generally older, included a higher proportion of females, and married. They also had fewer years of education (less than seven years), a lower than median annual household income (under 40 000 CNY), and a higher prevalence of overweight or obesity. For residential greenspace, we observed significant variations in NDVI values across our sample population. In 2018, annual average NDVI per 1 km radius buffer ranged from 0.08 to 0.42, with a median of 0.24 (IQR = 0.14). Respondents from the third quantile (0.24≤NDVI<0.29) experienced the lowest prevalence rate of multiple chronic conditions (7.9%) (**Table 1**).

**Table 1:** Baseline characteristics of the study population

Variables	Total	With multiple chronic conditions	With multiple chronic conditions	Cramer’s V	*P*-value*
Age				0.28	<0.01
*Below 45 y*	11 313	66 (0.2%)	11 247 (39.5%)		
*45 to 65 y*	11 804	1070 (3.8%)	10 734 (37.7%)		
*Above 65 y*	5371	1172 (4.1%)	4199 (14.7%)		
Sex				0.02	<0.01
*Male*	13 706	1028 (3.6%)	12 678 (44.5%)		
*Female*	14 782	1280 (4.5%)	13 502 (47.4%)		
Marital status				0.08	<0.01
*Never married*	2533	21 (0.1%)	2512 (8.8%)		
*Married and other*	25 955	2287 (8.0%)	2512 (8.8%)		
Education				0.16	<0.01
*<7 y of education*	9017	1335 (4.7%)	7682 (27.0%)		
*7 to 9 y of education*	10 078	606 (2.1%)	9472 (33.2%)		
*>9 y of education*	9393	416 (1.5%)	8977 (31.5%)		
Annual household income				0.23	<0.01
*≤40 000 CNY*	14 244	1474 (5.2%)	12 770 (44.8%)		
*>40 000 CNY*	14 244	834 (2.9%)	13 410 (47.1%)		
Body mass index				0.31	<0.01
*Under weight (<18.5)*	1348	89 (0.3%)	1259 (4.4%)		
*Normal weight (18.5–24.9)*	15 963	997 (3.5%)	14 966 (52.5%)		
*Overweight+ (≥25)*	11 177	1222 (4.3%)	9955 (34.9%)		
NDVI				0.22	<0.01
NDVI_Q1-1000m_ (0.081–0.151)	7278	591 (2.1%)	6687 (23.5%)		
NDVI_Q2-1000m_ (0.151–0.236)	7103	496 (1.7%)	6607 (23.2%)		
NDVI_Q3-1000m_ (0.236–0.290)	7147	568 (2.0%)	6579 (23.1%)		
NDVI_Q4-1000m_ (0.290–0.420)	6960	653 (2.3%)	6307 (22.1%)		

CNY – Chinese Yuan, NDVI – Normalized Difference Vegetation Index

*P-values were obtained under the χ^2^ test.

### Residential greenspace and multiple chronic conditions

For the fully adjusted model, all three quartiles (NDVI_Q2_, NDVI_Q3_, and NDVI_Q4_) where significantly associated with lower likelihood of experiencing multiple chronic conditions compared against the baseline NDVI_Q1_ quartile. The adjusted β coefficients and corresponding 95% CIs were: −0.30 (95% CI = −0.47, −0.13) for NDVI_Q2_, −0.37 (95% CI = −0.59, −0.16) for NDVI_Q3_, and −0.36 (95% CI = −0.59, −0.13) for NDVI_Q4_, respectively (**Table 2**). From the Poisson regression, we observed that higher exposure to residential greenspace was positively associated with lower number of chronic conditions. Compared against the baseline quartile NDVI_Q1_, adjusted β coefficients and 95% CIs were: −0.14 (95% CI = −0.22, −0.07) for NDVI_Q2_, −0.17 (95% CI = −0.27, −0.08) for NDVI_Q3_, and −0.11 (95% CI = −0.21, −0.01) for NDVI_Q4_, respectively (**Table 3**).

**Table 2:** Generalised linear mixed model results for multiple chronic conditions

Variables	β (95% CI)	*P*-value*
Sex (base = female)		
*Male*	−0.06 (−0.15, 0.03)	0.200
Age (base = <45 y old)		
*45–65*	2.56 (2.31, 2.80)	<0.001
*>65 y old*	3.55 (3.30, 3.81)	<0.001
Marriage (base = never married)		
*Married*	0.37 (−0.07, 0.80)	0.097
Region (base = urban)		
*Rural*	0.25 (0.10, 0.40)	<0.001
Annual household income (base = ≤40 000 CNY)		
*More than 40 000 CNY*	−0.14 (−0.25, −0.04)	0.007
Education (base = <7 y of education)		
*7 to 9 y of education*	−0.31 (−0.42, −0.18)	<0.001
*>9 y of education*	−0.14 (−0.29, −0)	0.054
BMI (base = normal weight)		
*Underweight*	0.02 (−0.22, 0.25)	0.888
*Overweight or obese*	0.64 (0.55, 0.74)	<0.001
NDVI (base = Q1)		
*Q2*	−0.30 (−0.47, −0.13)	<0.001
*Q3*	−0.37 (−0.59, −0.16)	<0.001
*Q4*	−0.36 (−0.59, −0.13)	<0.001

BMI – body mass index, CI – confidence interval, CNY – Chinese Yuan, NDVI – Normalized Difference Vegetation Index

*P-values were obtained under Wald z-tests.

**Table 3:** Poisson regression results for the number of chronic diseases

Variables	β (95% CI)	*P*-value*
Sex (base = female)		
*Male*	−0.02 (−0.06, 0.02)	0.330
Age (base = <45 y old)		
*45–65*	1.75 (1.67, 1.83)	<0.001
*>65 y old*	2.32 (2.24, 2.41)	<0.001
Marriage (base = never married)		
*Married*	0.44 (0.27, 0.60)	<0.001
Region (base = urban)		
*Rural*	0.11 (0.05, 0.18)	<0.001
Annual household income (base = ≤40 000 CNY)		
*More than 40 000 CNY*	−0.13 (−0.17, −0.08)	<0.001
Education (base = <7 y of education)		
*7 to 9 y of education*	−0.17 (−0.22, −0.12)	<0.001
*>9 y of education*	−0.16 (−0.23, −0.11)	<0.001
BMI (base = normal weight)		
*Underweight*	0.01 (−0.10, 0.10)	0.938
*Overweight or obese*	0.39 (0.36, 0.43)	<0.001
NDVI (base = Q1)		
*Q2*	−0.14 (−0.22, −0.07)	<0.001
*Q3*	−0.17 (−0.27, −0.08)	<0.001
*Q4*	−0.11 (−0.21, −0.01)	0.03

BMI – body mass index, CI – confidence interval, CNY – Chinese Yuan, NDVI – Normalized Difference Vegetation Index

*P-values were obtained under Wald z-tests.

### Association between residential greenspace and NCD classes

**Figure 1:**
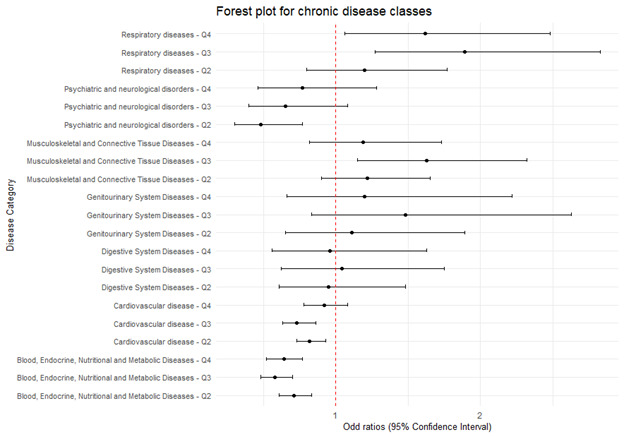
Association between greenspace and seven major chronic disease classes.

When examining the relationship across NCD classes, the evidence indicated that higher exposure to residential greenspace was associated with a reduced risk of experiencing blood, endocrine, nutritional and metabolic chronic diseases (**Figure 1**). We found no conclusive evidence for other NCD classes. Specifically, the adjORs and 95% CIs for blood, endocrine, nutritional and metabolic diseases were: 0.71 (95% CI = 0.61, 0.83) for NDVI_Q2_, 0.58 (95% CI = 0.48, 0.70) for NDVI_Q3_, and 0.64 (95% CI = 0.52, 0.77) for NDVI_Q4_, respectively, compared to baseline NDVI_Q1_. Additionally, exposure to low and medium levels of greenspace were associated with a reduced risk of CVD and mental illnesses. For CVD, the adjOR was 0.82 (95% CI = 0.73, 0.93) for NDVI_Q2_ and 0.73 (95% CI = 0.63, 0.86) for NDVI_Q3_. For psychiatric and neurological disorders, adjOR for NDVI_Q2_ was 0.48 (95% CI = 0.30, 0.77). Conversely, chronic respiratory diseases exhibited a positive association with greenspace exposure. Significant associations were observed for NDVI_Q3_ (adjOR = 1.89; 95% CI = 1.27, 2.83) and NDVI_Q4_ (adjOR = 1.62; 95% CI = 1.06, 2.48), whereas no significant association was found for NDVI_Q2_ (adjOR = 1.20; 95% CI = 0.80, 1.77). For musculoskeletal diseases, a significant association was found for NDVI_Q3_ (adjOR = 1.63; 95% CI = 1.15, 2.32). The associations for other categories of chronic diseases were not significant.

### Sensitivity analyses

Repeating the analysis using average NDVI measurements within a 1 km buffer from the 24- and 36-month periods prior to 2018 yielded results consistent with the base case, showing a similar association between greenspace exposure and multiple chronic conditions (Table S3 in **Online Supplementary Document**). This suggests the potential presence of a time-lagged effect. Additionally, similar results were evident under repeated analysis using a radius buffer of 2 km, 3 km and 4 km (Table S4 in **Online Supplementary Document**). However, the association was not statistically significant when using a 5 km NDVI buffer, with adjusted odds ratios of 0.94 (95% CI = 0.80, 1.10) for NDVI_Q2_, 0.86 (95% CI = 0.71, 1.05) for NDVI_Q3_, and 0.94 (95% CI = 0.76, 1.05) for NDVI_Q4_, respectively.

## DISCUSSION

Utilising high-resolution satellite imagery and self-reported health outcomes from the 6th HSS in Shandong Province, we performed a cross-sectional analysis to investigate the relationship between residential greenspace exposure and the prevalence of multiple chronic conditions. Our findings suggested that increased exposure to greenspace was significantly associated with a reduced risk of experiencing multiple chronic conditions. This beneficial relationship persisted across various spatial buffers and temporal analyses. Additionally, our analysis revealed that older adults, individuals with lower educational attainment, lower income, rural residents, and those with obesity were more susceptible to developing multiple chronic conditions. Furthermore, utilising the HSS's predefined chronic disease classes, we found that the beneficial relationship between greenspace exposure and health outcomes was most pronounced for blood, endocrine, nutritional and metabolic chronic diseases.

To our knowledge, this study is among the few that investigate the effects of greenspace exposure on multiple chronic conditions. Existing literature has predominantly focused on its relationship with individual chronic diseases or single-class conditions. For example, numerous studies have reported the beneficial effects of greenspace on obesity, anxiety, hypertension, diabetes and other chronic conditions [21–25]. A recent review found mixed results when examining over 100 health outcomes relating to greenspace exposure, including mortality, cardiovascular diseases, pregnancy outcomes, mental health, general health, allergic diseases, and blood biomarkers [14]. Examining the relationship for multiple chronic conditions, rather than focusing on single chronic diseases, may be meaningful because the mechanisms through which greenspace benefits health and well-being extend across physiological systems and disease categories. For example, one proposed pathway suggests that greenspace promotes physical activity, which has been demonstrated to improve various health outcomes, including cardiovascular and mental health [26–29]. Other studies have suggested that greenspace mitigates environmental risk factors, such as air pollution, noise, and heat, thereby, potentially contributing to lowering the risk for a range of NCDs [19,30,31].

Our findings contribute to existing research on the relationship between environmental factors and multimorbidity, expanding a literature base that has been predominantly focused on air pollution. For instance, Sun et al. investigated how residential proximity to major roadways contributes to multiple chronic diseases [32]. Numerous cross-sectional studies have explored the link between long-term exposure to air pollutants like particulate matter, nitrogen dioxide, and ozone, and the prevalence of multimorbid, multi-organ conditions [33–36]. Moreover, other longitudinal studies have demonstrated associations between higher concentrations of air pollution, climate change, and increased multimorbidity rates [37]. Despite numerous studies demonstrating that increased greenspace density can potentially mitigate air pollution and heat exposure, there is a notable lack of evidence regarding the relationship between greenspace and multimorbidity. One epidemiological study conducted across four southwestern Chinese provinces (Yunnan, Guizhou, Sichuan, Chongqing, and Tibet) suggested that greenspace significantly reduced the risk of over 22 chronic conditions [37]. Our research in Shandong further corroborates the beneficial effects of greenspace on multiple health conditions and physiological systems. Future research is needed to investigate the potential mediation and interactional effects between greenspace and other environmental stressors, such as air pollution and heat exposure, on multiple chronic health conditions. Additionally, further studies are needed to understand the underlying mechanisms through which greenspace exposure enhances the health outcomes of various organ systems.

Establishing evidence linking residential greenspace to multiple chronic conditions, compared to single health conditions, strengthens the case for environmental interventions aimed at increasing greenspace exposure. Such interventions could yield significant health and economic benefits given the potential for greenspace to support health across multiple physiological and psychological pathways. Research on greenspace and multimorbidity has significant implications for urban planning, health care policy, and community development. Integrating greenspaces into urban environments can enhance physical activity, reduce stress, improve air quality, and mitigate heat islands, all of which potentially contribute to reducing the burden of multiple chronic diseases [19]. Additionally, research has shown that ensuring equitable access to greenspaces across all socio-economic and demographic groups can address health disparities and promote environmental equality [38]. This is particularly important considering the rising prevalence of multiple chronic conditions, their complex patient-care needs, and the increasingly disproportionate burden they place on the health care system in China.

There were several limitations in this study which are notable. First, due to the inherent limitations of cross-sectional design, we were unable to establish causality in the relationship between greenspace exposure and multiple chronic conditions. Additionally, although we have rigorously tested our model, there remained the potential for unaccounted confounding variables which may influence the observed associations. Second, despite the HSS being conducted by trained interviewers, there remained a potential for reporting bias and selection bias, which could potentially skew our results. One important aspect is the reliability of using self-reported health data potentially leading to the misclassification or underreporting of health conditions. This could particularly influence responses for conditions associated with stigma, such as mental health disorders. Third, the reliability of using NDVI as a measurement of long-term greenspace exposure may be influenced by other built environmental factors. Research suggests that greenspace accessibility, which is not fully accounted for by greenspace density alone, plays a significant role in determining greenspace exposure [39,40]. Additionally, recent studies have highlighted greenspace morphology as an important factor in understanding the health effects of greenspace on NCDs, emphasising the need for further investigation [31]. Finally, in our subgroup analysis, we observed that exposure to medium and high levels of greenspace was associated with an increased risk of respiratory chronic diseases, contrary to prevailing epidemiological evidence. While the cross-sectional design of our study restricts causal inference, one possible explanation could lie in China’s urban planning policies, which often prioritise high vegetation density in urbanised areas. These regions are characterised by elevated levels of exposure to particulate matter from infrastructure and transport, which may contribute to adverse respiratory health outcomes. Further research is warranted to better understand this relationship.

## CONCLUSIONS

We found significant associations between residential greenspace and the prevalence and count of multiple chronic conditions, as well as specific condition clusters. These findings highlight the importance of integrating greenspace into urban planning and environmental public health strategies to improve overall health outcomes and reduce the burden of multiple chronic diseases.

## Additional material


Online Supplementary Document
