# Supplementary material for: Residential greenspace and multiple chronic health conditions in China: a cross-sectional study
Source: J Glob Health. 2025 Jul 25;15:04218. doi: 10.7189/jogh.15.04218 (PMC12290433; doi:10.7189/jogh.15.04218)

Supplement to: Wang S, Sun J, Xu Z, Di Tanna GL, Chen M, Downey L E, Jan S, Si L. Residential greenspace and multiple chronic health conditions in China: a cross-sectional study. J Glob Health. 2025;15:04218.

Figure S1. Flowchart of the Sample Selection Process

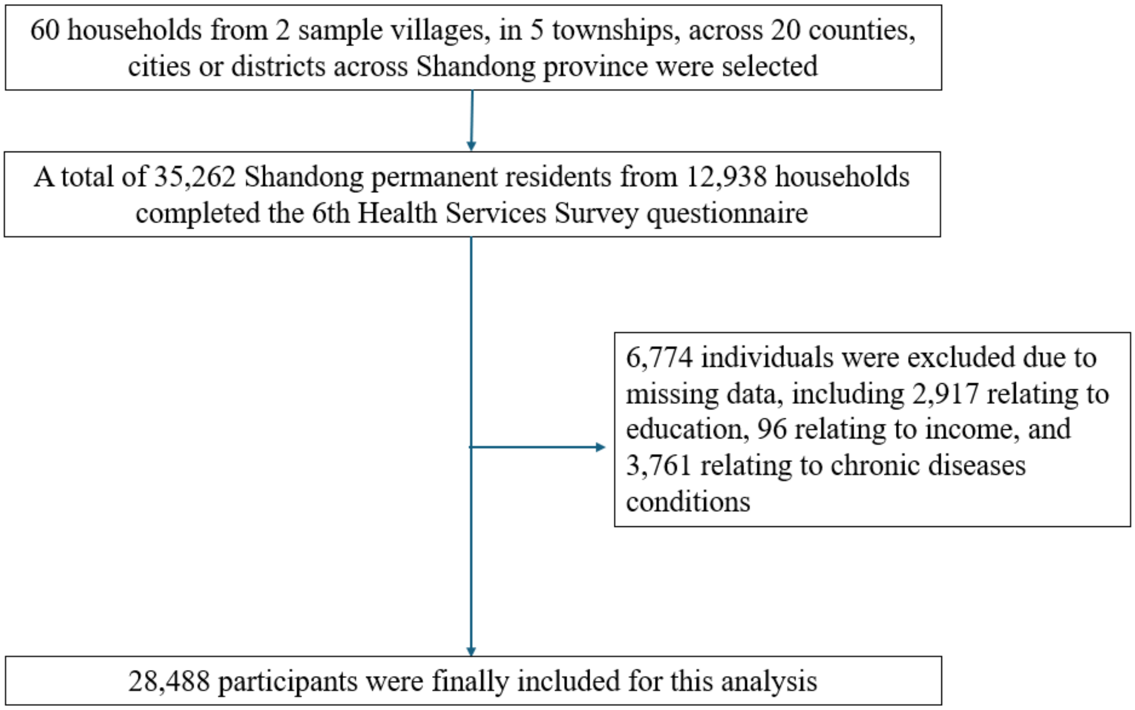

Table S1. Disease classification list for the Health Services Survey in Shandong province

A. Infectious disease
Hepatitis B
B. Cancer and tumors
Nasopharyngeal carcinoma
Esophageal cancer
Gastric cancer
Colorectal cancer
Rectal and anal cancer
Liver cancer
Pancreatic cancer
Tracheal, bronchial, and lung cancer
Breast cancer
Cervical cancer
Leukemia
Other types of cancer
Benign uterine tumor
Benign brain tumor
Other benign tumors
Carcinoma in situ
C. Endocrine, nutritional, metabolic diseases and immune disorders

Hyperthyroidism
Diabetes
Malnutrition
Rickets
Obesity and other nutritional disorders
Other endocrine, nutritional, metabolic, and immune disorders
D. Blood and hematopoietic organ diseases
Anemia
Other blood and hematopoietic organ diseases
E. Psychiatric disorders
Senile and pre-senile organic mental disorders
Schizophrenia
Depression
Other mental disorders
F. Neurological diseases
Meningitis
Epilepsy
Parkinson's disease
Other neurological disorders
G. Eye and adnexal diseases
Glaucoma
Cataract
Corneal diseases
Other eye and adnexal diseases
Ear and mastoid diseases
Otitis media and mastoiditis
Other ear and mastoid diseases
H. Circulatory system diseases
Chronic rheumatic heart disease
Other ischemic heart diseases
Pulmonary heart disease
Other types of heart disease
Hypertension
Cerebrovascular diseases
Varicose veins
Other circulatory system diseases
I. Respiratory system diseases
Pneumonia
Chronic pharyngitis and laryngitis
Emphysema
Other chronic obstructive pulmonary disease (COPD), including chronic bronchitis, etc.
Asthma
Other chronic respiratory system diseases
J. Digestive system diseases
Dental diseases
Other oral, salivary gland, and jaw diseases
Chronic gastroenteritis
Peptic ulcer
Abdominal hernia

Intestinal obstruction
Chronic liver disease
Liver cirrhosis
Cholelithiasis
Cholecystitis
Other Chronic digestive system disorders
K. Genitourinary disorders
Nephropathy
Nephritis
Pyelonephritis
Urinary tract stones
Other Chronic urogenital disorders
Benign prostatic hyperplasia or inflammation
Other chronic male genital disorders
Breast disease
Salpingitis
Oophoritis
Uterine prolapse
Other chronic female genital disorders
L. Skin and subcutaneous tissue disorders
Furuncle
Boil
Dermatitis
Other chronic skin and subcutaneous tissue disorders
M. Musculoskeletal and connective tissue disorders
Rheumatoid arthritis
Intervertebral disc disease
Osteomyelitis
Other chronic musculoskeletal disorders
N. Congenital disorders
Congenital heart disease
Other congenital abnormalities

Table S2. STROBE Statement—Checklist of items that should be included in reports of cross-sectional studies

	Item No	Recommendation	Reported in
**Title and abstract**	1	(*a*) Indicate the study's design with a commonly used term in the title or the abstract	P1
		(*b*) Provide in the abstract an informative and balanced summary of what was done and what was found	P2
**Introduction**			
Background/rationale	2	Explain the scientific background and rationale for the investigation being reported	P3
Objectives	3	State specific objectives, including any prespecified hypotheses	P3
**Methods**			
Study design	4	Present key elements of study design early in the paper	P4
Setting	5	Describe the setting, locations, and relevant dates, including periods of recruitment, exposure, follow-up, and data collection	P4
Participants	6	(*a*) Give the eligibility criteria, and the sources and methods of selection of participants	P4
Variables	7	Clearly define all outcomes, exposures, predictors, potential confounders, and effect modifiers. Give diagnostic criteria, if applicable	P5
Data sources/ measurement	8	For each variable of interest, give sources of data and details of methods of assessment (measurement). Describe comparability of assessment methods if there is more than one group	P5
Bias	9	Describe any efforts to address potential sources of bias	NA
Study size	10	Explain how the study size was arrived at	P4
Quantitative variables	11	Explain how quantitative variables were handled in the analyses. If applicable, describe which groupings were chosen and why	P4-5
Statistical methods	12	(*a*) Describe all statistical methods, including those used to control for confounding	P5

		(*b*) Describe any methods used to examine subgroups and interactions	P5
		(*c*) Explain how missing data were addressed	Supplementary Online Document
		(*d*) If applicable, describe analytical methods taking account of sampling strategy	P5
		(*e*) Describe any sensitivity analyses	P5
**Results**			
Participants	13*	(a) Report numbers of individuals at each stage of study—eg numbers potentially eligible, examined for eligibility, confirmed eligible, included in the study, completing follow-up, and analysed	Supplementary Online Document
		(b) Give reasons for non-participation at each stage	Supplementary Online Document
		(c) Consider use of a flow diagram	Supplementary Online Document
Descriptive data	14*	(a) Give characteristics of study participants (eg demographic, clinical, social) and information on exposures and potential confounders	P6
		(b) Indicate number of participants with missing data for each variable of interest	Supplementary Online Document
Outcome data	15*	Report numbers of outcome events or summary measures	P6
Main results	16	(*a*) Give unadjusted estimates and, if applicable, confounder-adjusted estimates and their precision (eg, 95% confidence interval). Make clear which confounders were adjusted for and why they were included	P6-7
		(*b*) Report category boundaries when continuous variables were categorized	P5
		(*c*) If relevant, consider translating estimates of relative risk into absolute risk for a meaningful time period	NA

Other analyses	17	Report other analyses done—eg analyses of subgroups and interactions, and sensitivity analyses	P7
**Discussion**			
Key results	18	Summarise key results with reference to study objectives	P7
Limitations	19	Discuss limitations of the study, taking into account sources of potential bias or imprecision. Discuss both direction and magnitude of any potential bias	P9
Interpretation	20	Give a cautious overall interpretation of results considering objectives, limitations, multiplicity of analyses, results from similar studies, and other relevant evidence	P7-9
Generalisability	21	Discuss the generalisability (external validity) of the study results	NA
**Other information**			
Funding	22	Give the source of funding and the role of the funders for the present study and, if applicable, for the original study on which the present article is based	P10

Table S3. Sensitivity analysis utilizing average NDVI measurements from 1km radius buffer from 24- and 36-month periods prior to 2018

**Variables**	**β (95% CI)**	**P value***
24-month NDVI averages prior to 2018 (base = $NDVI_{Q1}$)		
$NDVI_{Q2}$	-0.30 (-0.47, -0.13)	<0.001
$NDVI_{Q3}$	-0.44 (-0.66, -0.22)	<0.001
$NDVI_{Q4}$	-0.38 (-0.60, -0.15)	0.001
36-month NDVI averages prior to 2018 (base = $NDVI_{Q1}$)		
$NDVI_{Q2}$	-0.29 (-0.46, -0.11)	0.001
$NDVI_{Q3}$	-0.36 (-0.57, -0.15)	0.001
$NDVI_{Q4}$	-0.26 (-0.49, -0.04)	0.020

* P-values were obtained using Wald z-tests

Table S4. Sensitivity Analysis utilizing NDVI measurements from 2 km, 3 km, 4 km, and 5 km radius buffers and 24- and 36-month periods prior to 2018

**Variables**	**β (95% CI)**	**P value***
2km radius buffer in 2018 (base = $NDVI_{Q1}$)		
$NDVI_{Q2}$	-0.32 (-0.50, -0.14)	<0.001
$NDVI_{Q3}$	-0.53 (-0.76, -0.31)	<0.001
$NDVI_{Q4}$	-0.39 (-0.63, -0.15)	0.001
3km radius buffer in 2018 (base = $NDVI_{Q1}$)		

$NDVI_{Q2}$	-0.30 (-0.48, -0.12)	0.001
$NDVI_{Q3}$	-0.25 (-0.46, -0.03)	0.024
$NDVI_{Q4}$	-0.23 (-0.46, 0.01)	0.057
4km radius buffer in 2018 (base = $NDVI_{Q1}$)		
$NDVI_{Q2}$	-0.25 (-0.43, -0.06)	0.009
$NDVI_{Q3}$	-0.14 (-0.35, 0.07)	0.181
$NDVI_{Q4}$	-0.09 (-0.32, 0.14)	0.430
5km radius buffer in 2018 (base = $NDVI_{Q1}$)		
$NDVI_{Q2}$	-0.07 (-0.23, 0.10)	0.434
$NDVI_{Q3}$	-0.15 (-0.34, 0.05)	0.150
$NDVI_{Q4}$	-0.05 (-0.27, 0.16)	0.618
2km radius buffer in 2017 (base = $NDVI_{Q1}$)		
$NDVI_{Q2}$	-0.32 (-0.50, -0.14)	<0.001
$NDVI_{Q3}$	-0.57 (-0.80, -0.34)	<0.001
$NDVI_{Q4}$	-0.35 (-0.59, -0.11)	0.005
3km radius buffer in 2017 (base = $NDVI_{Q1}$)		
$NDVI_{Q2}$	-0.36 (-0.54, -0.18)	<0.001
$NDVI_{Q3}$	-0.46 (-0.68, -0.23)	<0.001
$NDVI_{Q4}$	-0.31 (-0.54, -0.07)	0.011
4km radius buffer in 2017 (base = $NDVI_{Q1}$)		
$NDVI_{Q2}$	-0.22 (-0.40, -0.03)	<0.001

$NDVI_{Q3}$	-0.36 (-0.59, -0.13)	<0.001
$NDVI_{Q4}$	-0.19 (-0.42, 0.05)	0.114
5km radius buffer in 2017 (base = $NDVI_{Q1}$)		
$NDVI_{Q2}$	-0.07 (-0.23, 0.01)	0.438
$NDVI_{Q3}$	-0.12 (-0.31, 0.07)	0.209
$NDVI_{Q4}$	-0.08 (-0.29, 0.13)	0.454
2km radius buffer in 2016 (base = $NDVI_{Q1}$)		
$NDVI_{Q2}$	-0.32 (-0.50, -0.14)	<0.001
$NDVI_{Q3}$	-0.52 (-0.74, -0.30)	<0.001
$NDVI_{Q4}$	-0.33 (-0.56, -0.10)	0.005
3km radius buffer in 2016 (base = $NDVI_{Q1}$)		
$NDVI_{Q2}$	-0.28 (-0.47, -0.09)	0.004
$NDVI_{Q3}$	-0.27 (-0.50, -0.05)	0.017
$NDVI_{Q4}$	-0.20 (-0.43, 0.04)	0.102
4km radius buffer in 2016 (base = $NDVI_{Q1}$)		
$NDVI_{Q2}$	-0.25 (-0.44, -0.07)	0.008
$NDVI_{Q3}$	-0.23 (-0.45, -0.01)	0.040
$NDVI_{Q4}$	-0.16 (-0.39, 0.07)	0.174
5km radius buffer in 2016 (base = $NDVI_{Q1}$)		
$NDVI_{Q2}$	0.01 (-0.16, 0.15)	0.937
$NDVI_{Q3}$	-0.18 (-0.38, 0.02)	0.077

$NDVI_{Q4}$	-0.06 (-0.27, 0.14)	0.542

* P-values were obtained using Wald z-tests

Figure S2. QQ plot and histogram of age

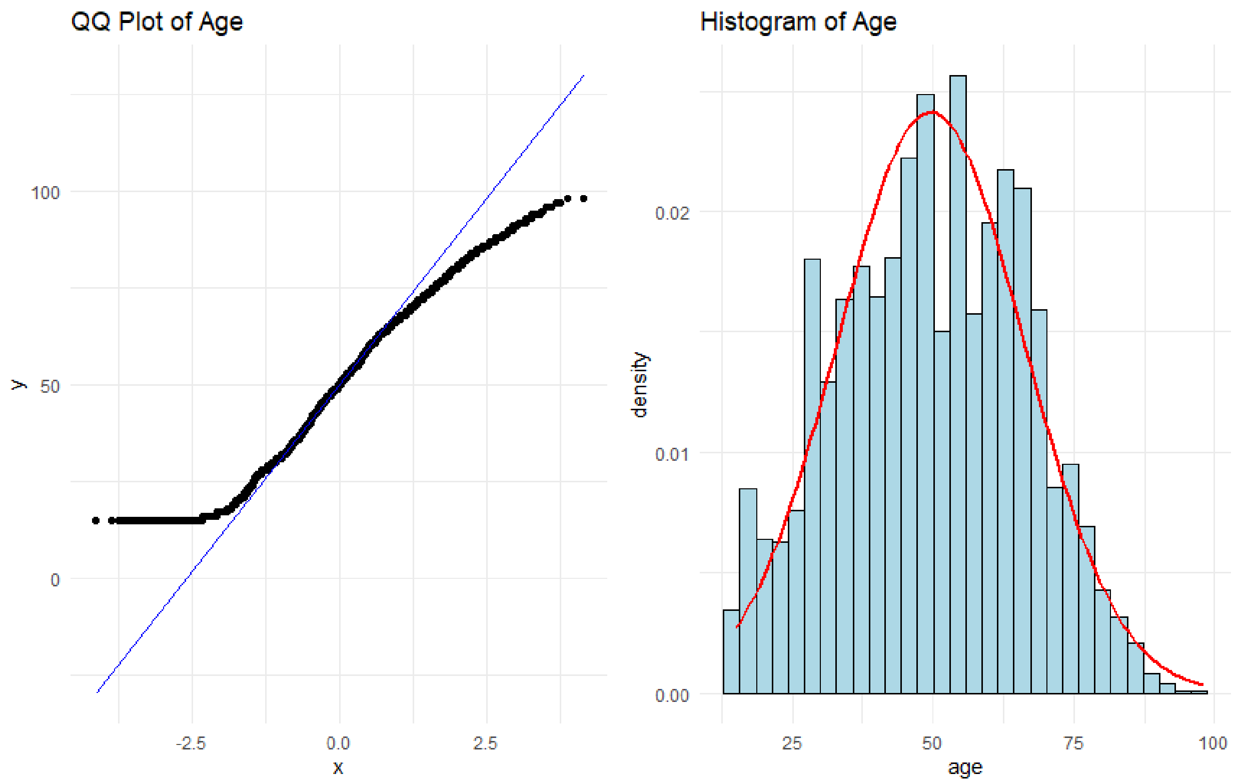

Figure S3. QQ plot and histogram of income

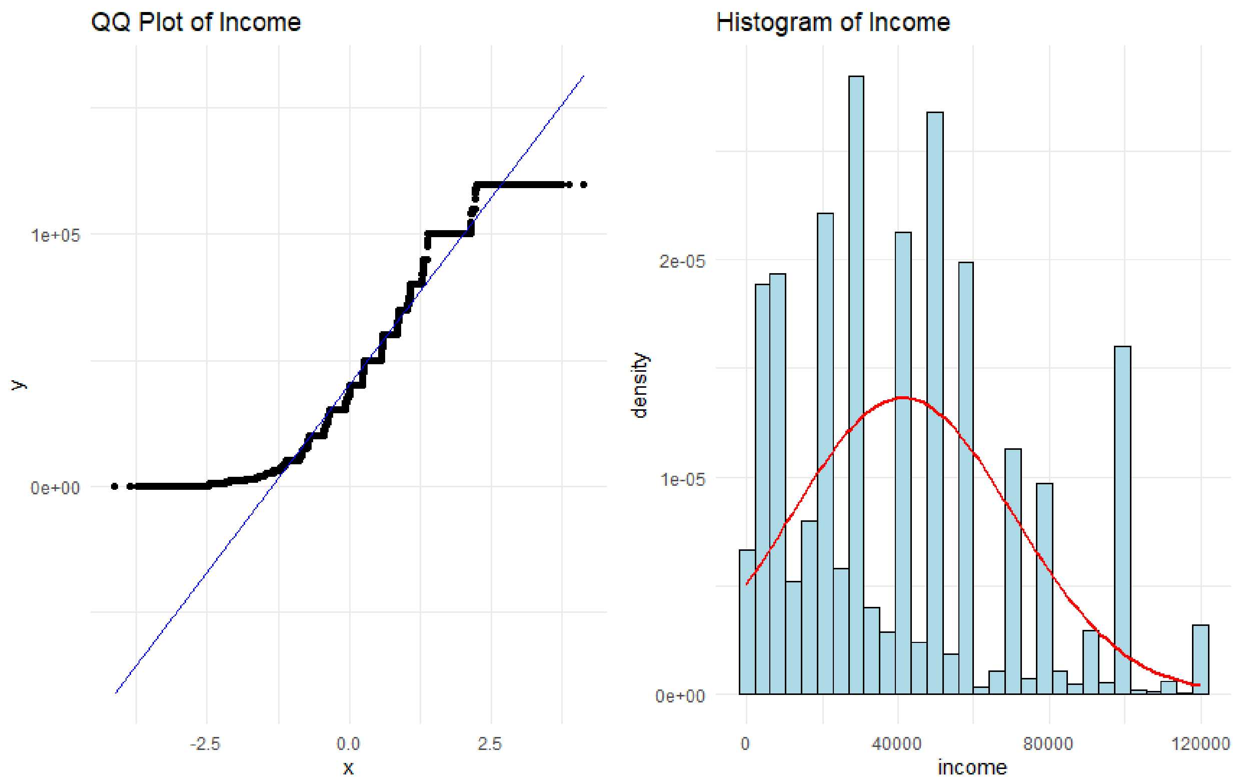

Figure S4. QQ plot and histogram of BMI

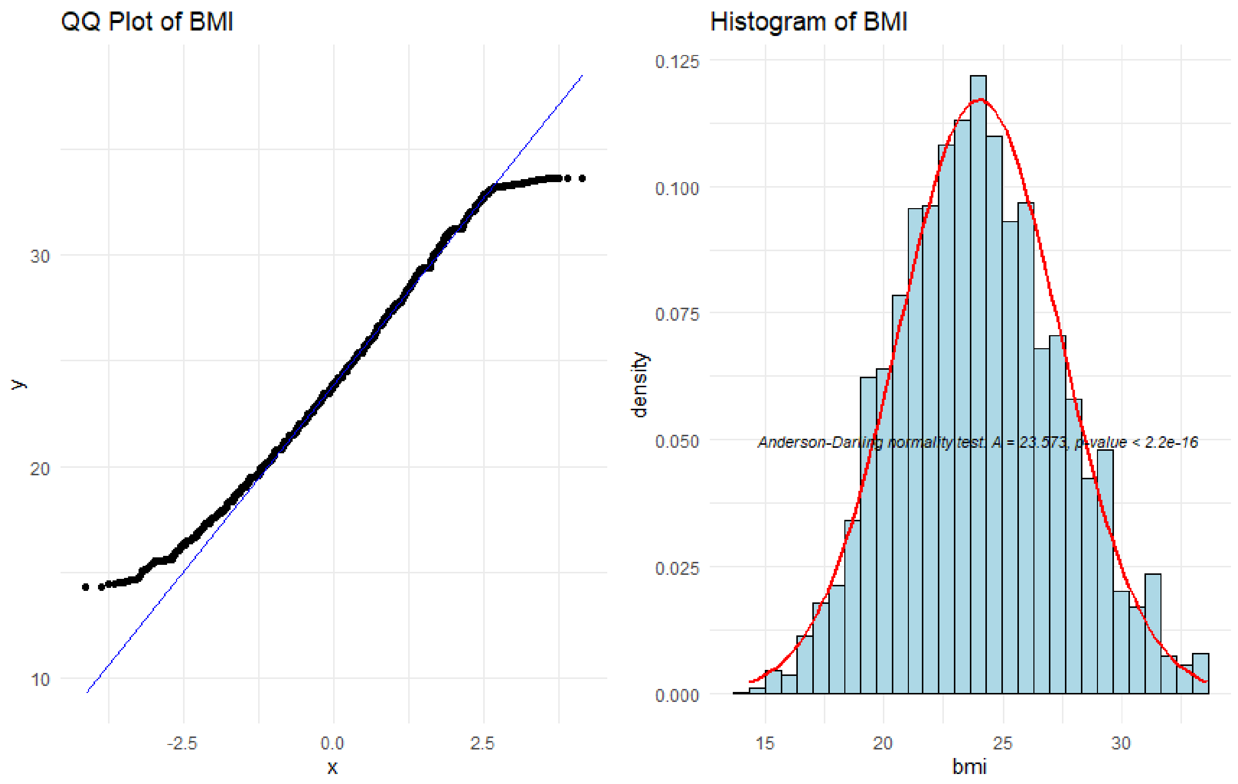

Figure S5. QQ plot and histogram of NDVI

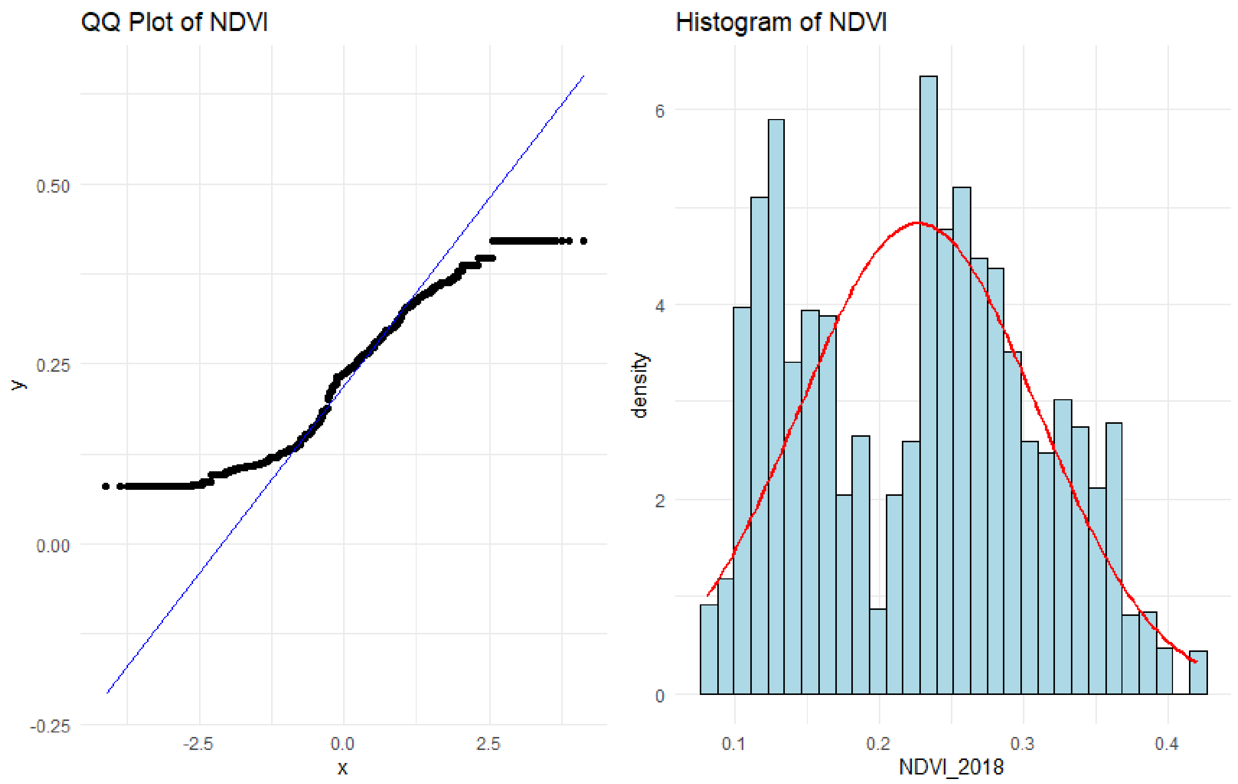

Supplement: Online Supplementary Document [file jogh-15-04218-s001.pdf]
